# The impact of aerobic exercise dose based on ACSM recommendations on patients with Parkinson’s disease: a systematic review and meta-analysis of randomized controlled trials

**DOI:** 10.3389/fnagi.2024.1419643

**Published:** 2024-10-03

**Authors:** Wenlai Cui, Zepeng Hu, Jian Li, Siji Wang, Ruilin Xu

**Affiliations:** ^1^School of Dance and Martial Arts, Capital University of Physical Education and Sports, Beijing, China; ^2^School of Kinesiology and Health, Capital University of Physical Education and Sports, Beijing, China; ^3^Institute of Physical Education and Training, Capital University of Physical Education and Sports, Beijing, China; ^4^School of Physical Education (Main Campus), Zhengzhou University, Zhengzhou, China

**Keywords:** Parkinson’s disease, aerobic exercise, dose, ACSM recommendation, meta-analysis

## Abstract

**Background:**

To explore the effects of different dose of aerobic exercise on motor function, balance, mobility, and quality of life in Parkinson’s disease patients, aiming to provide insights into determining the optimal aerobic exercise dose for treating PD.

**Methods:**

Searching was conducted in four databases: PubMed, Embase, Web of Science, and Cochrane. The dose of aerobic exercise intervention was evaluated based on the recommendations of the American College of Sports Medicine regarding the development and maintenance of cardiorespiratory health, muscle strength, and functional mobility in patients with PD. The exercise intervention dose of the included studies were first classified into high ACSM compliance and low ACSM compliance based on meeting 4/6 of the ACSM recommendations. The reliability of the results was then validated using the criterion of meeting 5/6 of the ACSM recommendations. Comparisons of the effects of aerobic exercise dose on Motor function, Balance, Mobility, and QOL in PD patients using standardized mean difference with 95% confidence intervals.

**Results:**

When using the 4/6 ACSM compliance criterion, 17 studies were categorized as high ACSM compliance and 12 as low ACSM compliance. The SMD ratios for high versus low ACSM compliance were: UPDRS-III (−0.79: −0.18), BBS (0.60: 0.05), TUG (−0.60: −0.60), and QOL (−1.05: −0.15). When using the 5/6 ACSM compliance criterion, 11 studies were categorized as high ACSM compliance and 19 as low ACSM compliance. The SMD ratios for high versus low ACSM compliance were: UPDRS-III (−0.95: −0.38), BBS (0.48: 0.37), TUG (−0.71: −0.55), and QOL (−0.7: 0.04).

**Conclusion:**

This study provides preliminary support for the potential of aerobic exercise to improve certain clinical symptoms in patients with PD. Furthermore, the results indicate that compliance to higher doses of aerobic exercise, as per ACSM standards, may contribute to improvements in motor function, balance, mobility, and quality of life for patients with PD. However, due to the heterogeneity in the studies and the influence of factors that have not yet been fully explored, these conclusions should be interpreted with caution. More high-quality randomized controlled trials are needed in the future to further verify and clarify the effects of aerobic exercise.

**Systematic review registration:**

https://www.crd.york.ac.uk/prospero, identifier PROSPERO: CRD42024517548.

## 1 Introduction

Parkinson’s disease (PD) is typically classified as a movement disorder (Tysnes and Storstein, 2017), primarily affecting the motor nervous system, with symptoms including muscle rigidity, tremors, bradykinesia, and gait abnormalities (Samii et al., 2004; Lew, 2007; Kalia and Lang, 2015). However, the detrimental impact of PD extends beyond motor impairment, often manifesting in cognitive, psychological, and sleep disturbances (Samii et al., 2004; Barnett-Cowan et al., 2010). On August 9, 2023, the World Health Organization reported a rapid increase in disability and mortality attributable to PD. Over the past 25 years, the prevalence of PD has doubled, with over 8.5 million global cases documented in 2019, resulting in 5.8 million disabilities and 329,000 deaths (World Health Organization, 2023). With the passage of time and shifts in global demographics, the trajectory of PD prevalence is expected to worsen further. Consequently, the exploration and application of therapies for PD assume growing importance.

PD currently remains incurable, with its etiology still not fully elucidated. The primary motor symptoms of PD arise from the degeneration of dopaminergic neurons in the substantia nigra of the midbrain, resulting in dopamine deficiency in relevant brain regions (Hughes et al., 1992; Reich and Savitt, 2019). Initial treatment of PD often involves the use of L-DOPA, with adjunct dopamine agonists utilized as its efficacy diminishes. As the disease progresses, there is typically a need to escalate medication dosages, which, unfortunately, may lead to the development of dyskinesias, primarily involuntary movements, when doses are increased (Sveinbjornsdottir, 2016). Levodopa/carbidopa is considered the most effective medication for symptom alleviation, functional improvement, and enhancement of quality of life (QOL), yet accessibility to it remains limited, particularly in low- and middle-income countries (Jankovic and Poewe, 2012). Despite the crucial role of pharmacotherapy and supportive therapies in managing PD symptoms (Zhu et al., 2022; Zhong et al., 2023), prolonged use of PD medications may engender a spectrum of adverse effects, including nausea, somnolence, and motor fluctuations, imposing additional burdens on patients’ lives (Samii et al., 2004; Jankovic and Poewe, 2012). In its latest guidelines on the treatment and care of PD, the World Health Organization specifically underscores the importance of exercise therapy. As a cost-effective rehabilitation intervention, exercise not only aids in improving the functionality and quality of life of PD patients but also alleviates the burden on caregivers (Bloem et al., 2015).

Practical research has revealed that regular exercise mitigates secondary musculoskeletal and cardiovascular issues stemming from reduced physical activity in patients with PD (Ascherio and Schwarzschild, 2016; Yu et al., 2022). Aerobic exercise, initially recognized for its cardiovascular benefits (Königstein et al., 2023), has garnered attention in the realm of neurologic disorders (Schirinzi et al., 2020; Rotondo et al., 2023). Researchers have shifted focus towards investigating the role of aerobic exercise in ameliorating motor function, QOL, and potential disease progression in PD patients (Liu et al., 2019; Stuckenschneider et al., 2019; Yu et al., 2022; Corcos et al., 2023). Michael F. Salvatore and colleagues explored the effects of aerobic exercise on early-stage PD patients. By establishing equivalency between human and animal models, they analyzed the impact of aerobic exercise dose on cardiovascular parameters and motor function in Pink1 gene knockout rats, revealing potential mechanisms for alleviating motor dysfunction in Parkinson’s disease (Salvatore et al., 2022). Additionally, numerous experimental studies have found that aerobic exercise, as a significant form of physical activity, can have positive effects on PD patients (Voss et al., 2011; Gaßner et al., 2022; Zhen et al., 2022).

In recent years, several meta-analysis studies have examined the effects of aerobic exercise on improving symptoms in PD. Marcos Paulo Braz de Oliveira et al. found that aerobic exercise notably promotes improvements in gait, functional capacity, and lower limb muscle strength in PD patients, although its impact on QOL was not significant (de Oliveira et al., 2021). Kai Zhen et al observed that aerobic exercise can enhance balance, gait, and motor function in PD patients (Zhen et al., 2022). Additionally, researchers have explored the effects of various aerobic exercise modalities such as walking, tai chi, and dance on PD patients (Carapellotti et al., 2020; Lei et al., 2022; Salse-Batán et al., 2022). Numerous studies suggest that moderate intensity and frequency of aerobic exercise may have positive effects on improving motor control, reducing symptom severity, and promoting neuroplasticity (Smith and Zigmond, 2003; Crizzle and Newhouse, 2006; Kim et al., 2023; Rotondo et al., 2023). However, there is currently no definitive exercise prescription for PD patients. In this context, delving into the influence of aerobic exercise dose on PD patients not only aids in optimizing exercise therapy formulation but also provides patients with more individualized and effective treatment strategies.

The American College of Sports Medicine (ACSM) recommendations refer to the guidelines and advice on exercise and physical fitness developed by the American College of Sports Medicine. These standards are based on scientific research and aim to optimize the development and maintenance of cardiorespiratory health, muscle strength, and functional mobility. Specifically, the ACSM recommendations include detailed advice on aerobic exercise, strength training, and flexibility exercises to help different populations formulate and follow effective exercise plans. Similarly, the ACSM has proposed aerobic exercise dose schemes for the rehabilitation of PD patients, including detailed descriptions of exercise intensity, duration, and frequency (Garber et al., 2011). Adhering to ACSM recommendations is associated with improved clinical outcomes, making them a suitable benchmark for evaluating the effectiveness of exercise interventions in PD patients. Therefore, the aim of this systematic review is to compare the effects of aerobic exercise interventions with high ACSM compliance versus low ACSM compliance on PD patients.

## 2 Methods

The systematic review and meta-analysis followed the Preferred Reporting Items for Systematic Reviews and Meta-Analyses (PRISMA) guidelines and were registered in PROSPERO (CRD42024517548).

### 2.1 Search strategy

Following the PICOS principles, searches were conducted in PubMed, Embase, Web of Science, and the Cochrane database, with the search period extending from the inception of the databases to February 24, 2024. P: The study population is patients with Parkinson’s disease (PD). I: The intervention for the experimental group is aerobic exercise. C: The control group receives conventional treatment. O: The outcomes are motor function, balance ability, mobility, and quality of life in PD patients. S: The study design is a randomized controlled trial. The search query is shown in Table 1 (using PubMed as an example). The specific search query can be found in Supplementary Appendix 1. Manual searches of bibliographies from relevant reviews and identified articles were performed to supplement the research. Contact with study authors was made when necessary to obtain additional information.

**TABLE 1:** Search strategy (Using PubMed as an example).

Steps	Search query
#1	Search: “Parkinson Disease” [Mesh] Sort by: Most Recent
#2	Search: ((((((((((Idiopathic Parkinson’s Disease [Title/Abstract]) OR (Lewy Body Parkinson’s Disease [Title/Abstract])) OR (Parkinson’s Disease, Idiopathic[Title/Abstract])) OR (Parkinson’s Disease, Lewy Body[Title/Abstract])) OR (Parkinson Disease, Idiopathic[Title/Abstract])) OR (Parkinson’s Disease[Title/Abstract])) OR (Idiopathic Parkinson Disease[Title/Abstract])) OR (Lewy Body Parkinson Disease[Title/Abstract])) OR (Primary Parkinsonism[Title/Abstract])) OR (Parkinsonism, Primary[Title/Abstract])) OR (Paralysis Agitans[Title/Abstract])
#3	#1 OR #2
#4	Search: “Exercise”[Mesh] Sort by: Most Recent
#5	Search: ((((((((((((Exercises[Title/Abstract]) OR (Sports[Title/Abstract])) OR (Physical Activity[Title/Abstract])) OR (Motor Activity[Title/Abstract])) OR (Training[Title/Abstract])) OR (endurance training[Title/Abstract])) OR (Tai Chi[Title/Abstract])) OR (yoga[Title/Abstract])) OR (Balance[Title/Abstract])) OR (Resistance[Title/Abstract])) OR (Flexibility[Title/Abstract])) OR (Cardiovascular[Title/Abstract])) OR (Aerobic[Title/Abstract])
#6	#4 OR #5
#7	Search: ((((Randomized controlled trial[Publication Type]) OR (controlled clinical trial[Publication Type])) OR (randomized[Title/Abstract])) OR (placebo[Title/Abstract])) OR (randomly[Title/Abstract])
#8	#3 AND #6 AND #7

### 2.2 Criteria for selection of studies

If studies met the following criteria, they were included:

(a) Published randomized controlled trials;
(b) Study participants diagnosed with PD;
(c) Experimental group intervention involving aerobic exercise;
(d) Control intervention could be no treatment or any treatment unrelated to exercise;
(e) Study outcome measures included Unified Parkinson’s Disease Rating Scale, Part III (UPDRS-III) or Movement Disorder Society Unified Parkinson’s Disease Rating Scale, Part III (MDS-UPDRS- III), Berg Balance Scale (BBS), Timed Up and Go (TUG) test, and 39-item Parkinson’s Disease Questionnaire (PDQ-39).

Exclusion criteria comprised:

(a) Animal model experiments, research reports, conference proceedings, reviews, etc.;
(b) Studies where the control group received exercise or studies lacking a conventional treatment control group;
(c) Studies involving participants with other cardiovascular or metabolic diseases;
(d) Studies in which participants received unconventional medication therapy during the exercise intervention were excluded;
(e) Duplicate experimental data from the same study in multiple publications.

Two authors (WLC and ZPH) independently screened titles and abstracts of identified literature for inclusion criteria. If either author deemed a study met the criteria, the full text was obtained. Subsequently, the full text was independently assessed by both authors for eligibility. In case of discrepancies, a third author (RLX) acted as an arbitrator, and consensus was reached through discussion. This study did not impose restrictions based on participant age, gender, publication date, or language.

### 2.3 Data synthesis and analysis

During the data extraction stage, two authors (WLC and JL) independently conducted operations using Excel spreadsheets to systematically organize the publication characteristics, methodological features, participant characteristics, exercise characteristics, risk assessment features, and outcome characteristics of the included studies. For studies presenting post-intervention outcome data in graphical form without clear textual explanations, we utilized Engauge Digitizer software for data extraction. In studies with multiple follow-ups, we only extracted the data from the first assessment after the intervention period ended.

Following data extraction, we evaluated the dose and compliance of exercise interventions. The evaluation of exercise intervention dose is based on ACSM recommendations for the development and maintenance of cardiorespiratory and neuromotor functions in PD (Gary et al., 2021). The ACSM recommended aerobic exercise dose is shown in Table 2 (11th Edition). Two authors (WLC and SJW) independently scored each study’s exercise intervention according to ACSM-recommended dose criteria, including frequency, intensity, and duration.

**TABLE 2:** Fitt recommendations for individuals with Parkinson’s disease (Aerobic exercise).

Load indicators	Content
Frequency	3–4d•wk^–1^
Intensity	High intensity [80–85% maximum heart rate (HRmax)] for mild-tomoderate Parkinson’s disease (PD); Moderate intensity (60–65% HRmax) for deconditioned individuals or those with more advanced PD; progress to 80–85% HRmax is possible.
Time	30 min of continuous or accumulated exercise.

Each scoring criterion for exercise indicators ranged from 0 to 2 points, where 2 points indicated compliance with the standard, 1 point indicated uncertainty, and 0 points indicated non-compliance. In cases of disagreement between two authors, consensus was reached through discussion with a third author. Using this scoring system, we calculated the proportion of studies adhering to the recommended exercise dose according to ACSM recommendations. Additionally, we used two classification criteria to ensure the reliability of the results. Studies with a proportion greater than or equal to 4/6 were classified as high compliance with ACSM recommendations, while those with a proportion less than 4/6 were classified as low compliance. To further validate the results, we revised the classification criteria, categorizing studies with a proportion greater than or equal to 5/6 as high compliance with ACSM recommendations, and those with a proportion less than 5/6 as low compliance.

### 2.4 Statistical analysis

We conducted meta-analysis using STATA 16.0 to compare the results of the included studies. The studies were categorized into two groups representing high compliance and low compliance with ACSM recommendations. The assessment of heterogeneity comprehensively considered the clinical characteristics of each study (e.g., participant characteristics, interventions, follow-up duration) and methodological features (e.g., study design, randomization methods, measurement tools). Based on this, we determined the model selection according to the degree of heterogeneity. If there was significant clinical or methodological heterogeneity, we used a random-effects model for effect size analysis; if the studies were relatively consistent in terms of clinical and methodological aspects, a fixed-effects model was applied. Additionally, we calculated the Higgins I^2^ statistic as an auxiliary tool for assessing statistical heterogeneity. Although the I^2^ value provides a quantitative measure of result variability, the primary criterion for model selection was clinical and methodological heterogeneity (Deeks et al., 2019). Effect sizes were expressed as standardized mean differences (SMD) with 95% confidence intervals (CI).

We constructed a funnel plot to assess publication bias and other types of small-study bias, as well as to visualize study heterogeneity. Through visual representation of the distribution of study results, we aimed to identify potential systematic biases. We employed Begg’s rank correlation test and Egger’s linear regression test to assess the asymmetry of funnel plots, setting p < 0.05 as statistically significant. Additionally, we conducted sensitivity analyses with stepwise exclusion of studies to validate the robustness of the findings. This series of statistical methods and sensitivity analyses were employed to ensure the reliability and accuracy of the meta-analysis, while providing a comprehensive assessment of heterogeneity among studies and potential publication bias.

### 2.5 Quality appraisal

Two pairs of authors (WLC and JL, ZPH and SJW) conducted a methodological quality assessment of the included literature using the quality assessment criteria recommended for randomized controlled trials by the Cochrane systematic review standards (Higgins et al., 2011). This review utilized the revised version of the Cochrane tool, known as the Risk of Bias tool (Rob 2), for the methodological quality assessment of the included studies (Sterne et al., 2019). The Rob 2 tool provides a framework for assessing the risk of bias in individual outcomes within any type of randomized trial. The assessment criteria include random sequence generation, allocation concealment, blinding of participants and personnel, blinding of outcome assessment, incomplete outcome data, selective reporting, and other biases, with reviewers scoring different studies based on the Cochrane Handbook (Cumpston et al., 2019).

## 3 Results

### 3.1 Study selection

**FIGURE 1:**
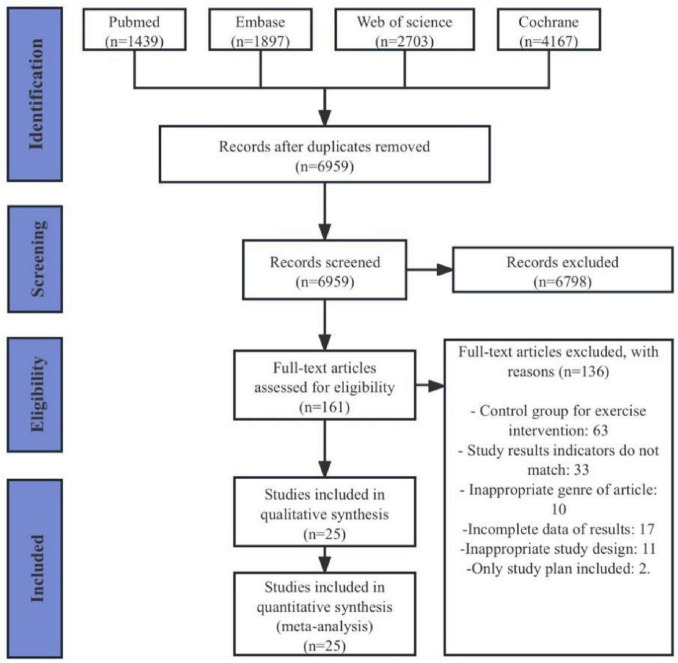
PRISMA flowchart of study selection.

A total of 10,206 articles were retrieved from the four databases (PubMed 1,439 articles, Embase 1,897 articles, Web of Science 2,703 articles, Cochrane 4,167 articles). After removing duplicates, 6,959 articles remained. Following the review of titles and abstracts, 161 articles were retained. After a thorough examination of the full texts, a final selection of 25 articles was included (Fisher et al., 2008; Schenkman et al., 2012; Amano et al., 2013; Gao et al., 2014; Conradsson et al., 2015; Cugusi et al., 2015; Liao et al., 2015; Collett et al., 2017; Demonceau et al., 2017; Ribas et al., 2017; Peloggia Cursino et al., 2018; Arfa-Fatollahkhani et al., 2019; Santos et al., 2019; Silva and Israel, 2019; Khuzema et al., 2020; Leavy et al., 2020; Moon et al., 2020; Youm et al., 2020; Göz et al., 2021; Khobkhun et al., 2021; Çoban et al., 2021; Li Z. et al., 2022; Li F. et al., 2022; Gulcan et al., 2023; Han et al., 2023; Figure 1).

### 3.2 Study characteristics

The characteristics of the included studies are presented in Table 3. The 25 selected articles encompassed 29 comparative studies, with 4 articles including two exercise intervention groups. The intervention groups comprised a total of 549 participants, while the control groups included 521 participants. One study did not report the gender ratio of participants; however, in the intervention group, there were 322 males and 236 females, while in the control group, there were 328 males and 206 females. The age range of participants in both intervention and control groups was similar, ranging from 40 to 80 years. There was a broad range in the duration of disease for participants in both groups, approximately 0.59 to 30 years. One studies did not report Hoehn and Yahr stage scores; however, the difference in Hoehn and Yahr stage scores between the two groups was not substantial. In terms of geographical distribution, four studies were from Brazil, four from the United States, six from China, four from Turkey, and two each from Sweden, India, and South Korea. There were also one study each from Belgium, Thailand, the United Kingdom, Iran, and Italy. Regarding participant recruitment, subjects were primarily recruited through hospital clinics, communities, and advertising media.

**TABLE 3:** Basic characteristics of the study.

Study	Country	Sample size	Gender Ratio (M/F)		Age (years)		Duration of disease		Hoehn AND Yahr		Medication status during the intervention period	Outcome
		**IG/CG**	**IG**	**CG**	**IG**	**CG**	**IG**	**CG**	**IG**	**CG**		
Liao et al., 2015	China	12/12	6/6	5/7	67.3 ± 7.1	64.6 ± 8.6	7.9 ± 2.7	6.4 ± 3.0	2.0 ± 0.7	1.9 ± 0.8	②	TUG, PDQ-39
Liao et al., 2015	China	12/12	6/6	5/7	65.1 ± 6.7	64.6 ± 8.6	6.9 ± 2.8	6.4 ± 3.0	2.0 ± 0.8	1.9 ± 0.8	②	TUG, PDQ-39
Conradsson et al., 2015	Sweden	47/44	28/19	22/22	72.9 ± 6.0	73.6 ± 5.3	6.0 ± 5.1	5.6 ± 5.0	2 or 3	2 or 3	①	UPDRS III
Khobkhun et al., 2021	Thailand	10/8	6/4	4/4	68.60 ± 6.67	68.88 ± 6.73	5.08	4.8	2.6	2.58	②	UPDRS III, TUG
Amano et al., 2013	US	15/9	7/8	7/2	66 ± 11	66 ± 7	8 ± 5	5 ± 3	2.4 ± 0.6	2.4 ± 0.4	⑤	UPDRS III
Fisher et al., 2008	US	10/10	6/4	8/2	64.0 ± 14.5	63.1 ± 11.5	14.7 ± 9.9	17.7 ± 13.3	1.9 ± 0.5	1.9 ± 0.3	②	UPDRS III
Collett et al., 2017	UK	54/51	31/23	30/21	66 ± 9	67 ± 7	4.8 ± 4.1	5.3 ± 4.1	NR	NR	②	UPDRS III
Leavy et al., 2020	Sweden	61/56	28/33	34/22	70 ± 8.5	70 ± 6.5	6.6 ± 5.1	8 ± 5.8	2–3	2–3	②	TUG
Peloggia Cursino et al., 2018	Brazilian	7/7	NR	NR	63.29 ± 11.06	72 ± 10.52	2.86 ± 1.57	6.29 ± 3.35	2 ± 0.82	2.29 ± 0.95	⑥	PDQ-39
Youm et al., 2020	South Korea	10/7	6/4	4/3	68.0 ± 6.8	72.1 ± 6.0	6.4 ± 3.6	8.0 ± 4.0	1–3	1–3	①	UPDRS III, TUG
Schenkman et al., 2012	US	31/31	26/15	26/15	63.4 ± 11.2	66.3 ± 10.1	3.9 ± 4.2	4.5 ± 3.8	2.2 ± 0.5	2.3 ± 0.4	①	UPDRS III, PDQ-39
Schenkman et al., 2012	US	33/31	24/15	26/15	64.5 ± 10.0	66.3 ± 10.1	4.9 ± 3.7	4.5 ± 3.8	2.3 ± 0.4	2.3 ± 0.4	①	UPDRS III, PDQ-39
Santos et al., 2019	Brazilian	13/14	11/2	11/3	61.7 ± 7.3	64.5 ± 9.8	7.0 ± 2.8	6.5 ± 2.0	1.4 ± 0.6	1.3 ± 0.3	NR	UPDRS III, TUG, PDQ-39
Demonceau et al., 2017	Belgium	16/15	12/4	10/5	65 ± 8	63.3 ± 6	5 ± 3	5 ± 2	1.75 ± 0.75	1.75 ± 0.25	NR	PDQ-39
Arfa-Fatollahkhani et al., 2019	Iran	11/9	8/3	7/2	60.63 ± 9.36	61.55 ± 8.57	8.89 ± 5.14	8.50 ± 6.34	2.13 ± 0.32	2.0 ± 0.35	③	TUG
Gao et al., 2014	China	37/39	23/14	27/12	69.54 ± 7.32	68.28 ± 8.53	9.15 ± 8.58	8.37 ± 8.24	1–3	1–3	NR	UPDRS III, BBS, TUG
Göz et al., 2021	Turkey	6/6	0/6	3/3	64 ± 15	61 ± 15	4.21 ± 0.96	5.5 ± 4.5	1.75 ± 0.25	1.5 ± 0.5	NR	BBS
Göz et al., 2021	Turkey	8/6	2/6	3/3	65 ± 13	61 ± 15	2.57 ± 2.44	5.5 ± 4.5	1 ± 0.5	1.5 ± 0.5	NR	BBS
Silva and Israel, 2019	Brazilian	14/11	8/6	6/5	63.12 ± 13,61	64.23 ± 13.45	NR	NR	3 ± 1	3 ± 1	④	BBS, TUG
Moon et al., 2020	South Korea	8/7	5/3	5/2	63.38 ± 5.37	62.14 ± 5.55	0.95 ± 0.36	0.96 ± 0.38	2.63 ± 0.52	2.71 ± 0.49	①	BBS TUG
Cugusi et al., 2015	Italy	10/10	8/2	8/2	68.1 ± 8.7	66.6 ± 7.3	7 ± 2	7 ± 4	2.4 ± 0.8	2.3 ± 0.5	NR	UPDRS III, BBS, TUG
Ribas et al., 2017	Brazilian	10/10	4/6	4/6	61.70 ± 6.83	60.20 ± 11.29	6.5 ± 4	7 ± 2.79	1.5 ± 0.5	1.75 ± 0.25	②	BBS
Khuzema et al., 2020	India	9/9	6/3	7/2	72 ± 5.22	70.89 ± 6.01	5.67 ± 2.33	5.23 ± 3.12	2.5–3	2.5–3	NR	BBS, TUG
Khuzema et al., 2020	India	9/9	6/3	7/2	68.11 ± 4.23	70.89 ± 6.01	6.2 ± 1.67	5.23 ± 3.12	2.5–3	2.5–3	NR	BBS, TUG
Çoban et al., 2021	Turkey	20/20	9/11	10/10	58.85 ± 8.09	60.75 ± 7.62	5.32 ± 6.23	5.35 ± 3.33	2.05	2.3	④	BBS TUG
Li Z. et al., 2022	China	20/20	13	16/4	67.57 ± 3.95	70 ± 5.59	6.83 ± 4.09	7.76 ± 4.55	1–3	1–3	③	UPDRS III, TUG, PDQ-39
Li F. et al., 2022	China	17/19	8	9/10	67.40(6.06)	69.16(6.17)	3.65(3.37)	4.89 ± 3.53	1–3	1–3	②	UPDRS III, BBS, TUG
Han et al., 2023	China	24/24	12	11/13	58.17 ± 5.36	56.25 ± 4.82	1.71 ± 0.75	1.83 ± 0.70	1.33 ± 0.48	1.38 ± 0.49	②	BBS, TUG
Gulcan et al., 2023	Turkey	15/15	13	13/2	61 ± 6.6667	60 ± 11.852	6 ± 6.6667	6 ± 5.9259	3 ± 0.7407	3 ± 0.7407	①	UPDRS III, BBS, TUG

IG, intervention group; CG, control group; M, male; F, female; NR, no report, ① Standard treatment, ② Stable medication therapy, ③ Exclusion of inconsistent medication use, ④ Exclusion of subjects with changes in medication intake parameters during the study, ⑤ No additional treatment received, ⑥ carry out the evaluations and interventions in phase “on” drugs for PD.

The intervention characteristics of the included studies are outlined in Table 4. All studies involved aerobic exercise interventions, including balance training, treadmill training, and Tai Chi, among others. In terms of outcome measures, 14 studies included UPDRS-III, involving a total of 630 participants (322 in the experimental group, 308 in the control group). Thirteen studies included BBS, with 372 participants (187 in the experimental group, 185 in the control group). Eighteen studies included TUG, with 613 participants (312 in the experimental group, 301 in the control group). Eight studies included a measurement of QOL, involving 282 participants (144 in the experimental group, 142 in the control group).

**TABLE 4:** Study intervention and outcome reporting characteristics.

Study	Interventions	Length of intervention	Frequency	Points	Intensity	Points	Duration	Points	ACSM compliance	
									(4/6)	(5/6)
Liao et al., 2015	Virtual Reality–Based Wii Fit Exercise [yoga exercises (10 min), strengthening exercises (15 min), balance games (20 min), treadmill (15min)]	6 weeks	2	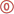	NR	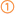	60 min	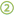	LOW	LOW
Liao et al., 2015	Traditional exercise [stretching exercises (10 min), strengthening exercises (15 min), balance exercises (20 min)]	6 weeks	2	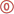	NR	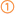	60 min	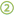	LOW	LOW
Conradsson et al., 2015	HiBalance program	10 weeks	3	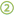	High intensity	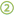	60 min	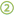	HIGH	HIGH
Khobkhun et al., 2021	Home-based exercise program [segmental rotation exercise in supine (45 min), throwing task (5 min), gait training (10 min)]	10 weeks	7	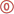	NR	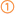	55 min	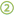	LOW	LOW
Amano et al., 2013	Tai Chi exercise	16 weeks	2	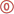	NR	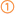	60 min	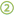	LOW	LOW
Fisher et al., 2008	Body weight-supported treadmill training	8 weeks	3	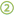	ACSM	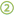	45 min	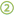	HIGH	HIGH
Collett et al., 2017	Aerobic and resistance training	12months	2	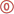	55–85%HRmax	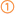	30 min	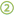	LOW	LOW
Leavy et al., 2020	HiBalance program	10 weeks	2	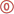	NR	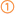	60 min	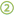	LOW	LOW
Peloggia Cursino et al., 2018	Partial body weight-supported treadmill training	6 weeks	3	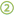	NR	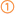	30 min	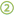	HIGH	HIGH
Youm et al., 2020	Progressive trunk resistance and stretching exercise	12 weeks	3	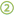	RPE: 2–6	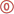	60–90 min	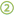	HIGH	LOW
Schenkman et al., 2012	Aerobic exercise program (using a treadmill, bike, or elliptical trainer)	16months	3	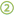	65–80%HRmax	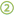	40–60 min	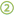	HIGH	HIGH
Schenkman et al., 2012	Flexibility/Balance/Function Exercise program (individualized spinal and extremity exibility exercises followed by group balance/functional training)	16months	3	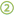	NR	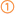	40–60 min	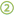	HIGH	HIGH
Santos et al., 2019	Nintendo Wii	2months	2	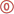	Ind. tail	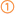	40 min	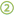	LOW	LOW
Demonceau et al., 2017	Aerobic training	12 weeks	2–3	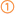	40–80%PWL	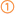	60–90 min	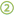	HIGH	LOW
Arfa-Fatollahkhani et al., 2019	Treadmill training	10 weeks	2–3	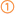	60%HRR	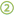	30 min	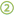	HIGH	HIGH
Gao et al., 2014	24-form Yang style Tai Chi	12 weeks	3	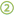	NR	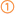	60 min	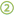	HIGH	HIGH
Göz et al., 2021	Pilates exercise	6 weeks	2	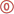	NR	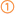	60 min	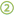	LOW	LOW
Göz et al., 2021	Elastic taping exercise	6 weeks	2	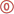	NR	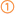	60 min	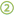	LOW	LOW
Silva and Israel, 2019	Dual-task aquatic exercise	10 weeks	2	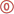	Moderate to high intensity	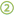	40 min	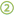	HIGH	LOW
Moon et al., 2020	Wii fit balance training	8 weeks	3	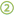	NR	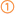	30 min	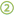	HIGH	HIGH
Cugusi et al., 2015	Nordic walking (a warm up period, practicing NW competence, improving intensity and distance of NW, and finally a cool down period)	12 weeks	2–3	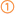	60%–80%HRR	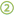	60 min	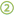	HIGH	HIGH
Ribas et al., 2017	Exergaming (Table Tilt, Tilt City, penguin slide, soccer heading, basic run, obstacle course and basic step)	12 weeks	2	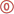	NR	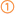	30 min	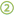	LOW	LOW
Khuzema et al., 2020	Tai Chi exercise	8 weeks	5	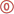	RPE: 11–15	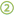	30–40 min	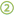	HIGH	LOW
Khuzema et al., 2020	Yoga exercise	8 weeks	5	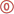	RPE: 11–15	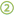	30–40 min	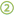	HIGH	LOW
Çoban et al., 2021	Pilates exercise	8 weeks	2	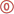	Ind. tail	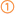	45min	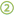	LOW	LOW
Li Z. et al., 2022	Wuqinxi Qigong exercise	12 weeks	2	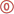	60–70%HRmax	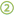	90 min	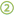	HIGH	LOW
Li F. et al., 2022	Yang-ge dancing	4 weeks	5	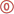	Moderate to high intensity	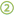	60 min	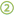	HIGH	LOW
Han et al., 2023	Visual feedback balance training	4 weeks	5	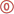	NR	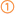	20 min	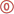	LOW	LOW
Gulcan et al., 2023	Augmented and virtual reality gait training	6 weeks	3	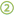	Moderate to high intensity	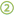	90 min	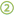	HIGH	HIGH

IG, intervention group; CG, control group; M, male; F, female; TAU, treatment as usual; HRR, heart rate reserve; VO^2^R, oxygen uptake reserve; RPE, rating of perceived exertion; HRmax, maximum heart rate; PWL, peak work load; Ind. Tail, individually tailored; NR, not reported.

The intervention durations ranged from 6 weeks to 16 months, with exercise frequencies varying from 2 times per week to 7 times per week. The duration of each exercise session ranged from 20 minutes to 90 minutes. When using the 4/6 ACSM compliance criterion, 17 studies were categorized as high ACSM compliance and 12 as low ACSM compliance. When using the 5/6 ACSM compliance criterion, 11 studies were categorized as high ACSM compliance and 19 as low ACSM compliance.

### 3.3 Risk of bias

**FIGURE 2:**
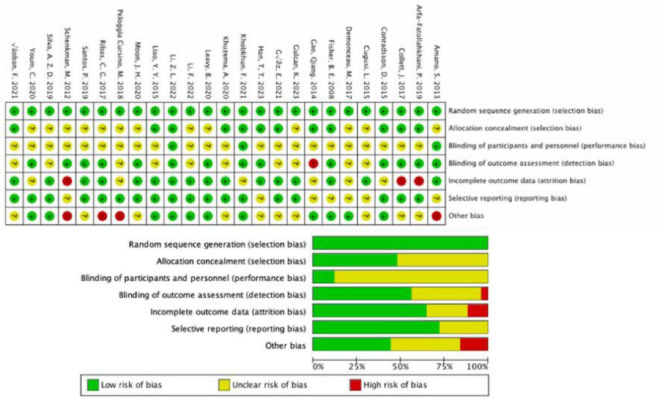
Results of Cochrane risk of bias tool. Above: Risk of bias summary: review authors’ judgments about each risk of bias item for each included study. Below: Risk of bias graph: review authors’ judgments about each risk of bias item presented as percentages across all included studies.

All studies uniformly indicated a low risk of bias in random sequence generation. In 12 studies, the risk of bias for allocation concealment was considered low, while in 13 studies, there was an uncertain risk of bias due to the lack of reported allocation methods. The assessment of blinding for both researchers and participants revealed a higher risk of bias, as the implementation of intervention training became complex under double-blind conditions, thus overall increasing the bias risk for this criterion. Regarding outcome assessment blinding, 14 studies employed random testing or had assessments conducted by blinded assessors, indicating a low risk. Eleven studies did not explicitly mention the outcome assessment method, posing some risk, while one study failed to ensure outcome blinding, resulting in a high risk. For incomplete outcome reporting, 16 studies had consistent or nearly consistent participant numbers post-intervention compared to baseline, indicating a low risk. Six studies had a relatively small number of participant withdrawals (5–10 individuals), suggesting some risk, while three studies had a large discrepancy in participant numbers before and after (greater than or equal to 10 individuals), indicating a high risk. In terms of selective reporting bias, 18 studies had a low risk, as they did not selectively report outcomes. Seven studies posed some risk because they did not report pre-registered protocols or provide detailed explanations for participant withdrawals. Additionally, four studies had a risk of other biases (Figure 2).

### 3.4 Meta-analysis

#### 3.4.1 Motor function

**FIGURE 3:**
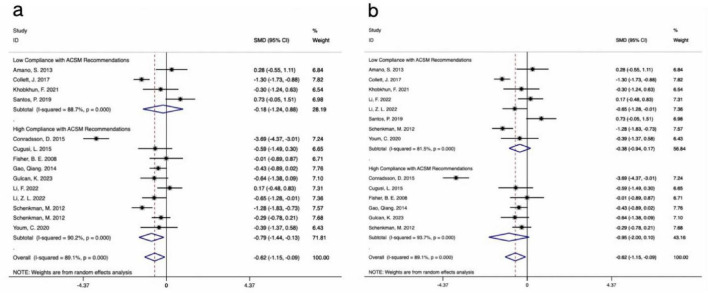
Forest plot of meta-analysis on the effect of exercise dose on UPDRS-III in PD patients [ACSM compliance classification criteria: **(a)**, 4/6; **(b)**, 5/6].

The results of 14 studies, which included UPDRS-III data as outcome measures, were analyzed. A comprehensive assessment of the clinical and methodological characteristics of the included studies was conducted, and a random effects model was used for analysis. The meta-analysis indicated that aerobic exercise intervention was more effective in improving UPDRS-III scores in PD patients compared to the control group [SMD, −0.62, (95% CI, −1.15 to −0.09), I^2^ = 89.1%]. A subgroup analysis based on the 4/6 ACSM compliance criterion revealed that, compared to low ACSM compliance, high ACSM compliance in aerobic exercise dose showed a greater improvement in the UPDRS-III score for PD patients [High Compliance with ACSM Recommendations: SMD, −0.79, (95% CI, −1.44, −0.13), I^2^ = 90.2%; Low Compliance with ACSM Recommendations: SMD, −0.18, (95% CI, −1.24, 0.88), I^2^ = 88.7%] (Figure 3a). A subgroup analysis based on the 5/6 ACSM compliance criterion also showed that high ACSM compliance in aerobic exercise dose resulted in greater improvement in the UPDRS-III score for PD patients [High Compliance with ACSM Recommendations: SMD, −0.95, (95% CI, −2.00, 0.1), I^2^ = 93.7%; Low Compliance with ACSM Recommendations: SMD, −0.38, (95% CI, −0.94, 0.17), I^2^ = 81.5%] (Figure 3b).

**FIGURE 4:**
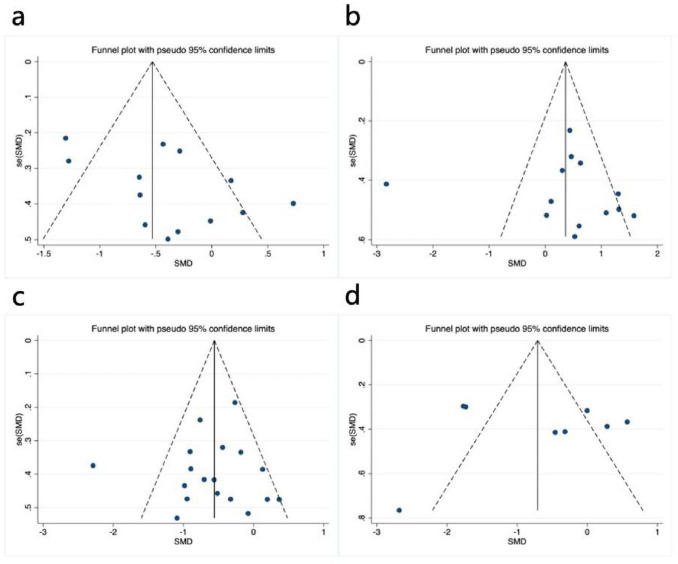
Funnel plot of meta-analysis on the effect of exercise dose on UPDRS-III, BBS, TUG, and PDQ-39 in PD patients. **(a)** UPDRS-III, **(b)** BBS, **(c)** TUG, **(d)** PDQ-39.

**FIGURE 5:**
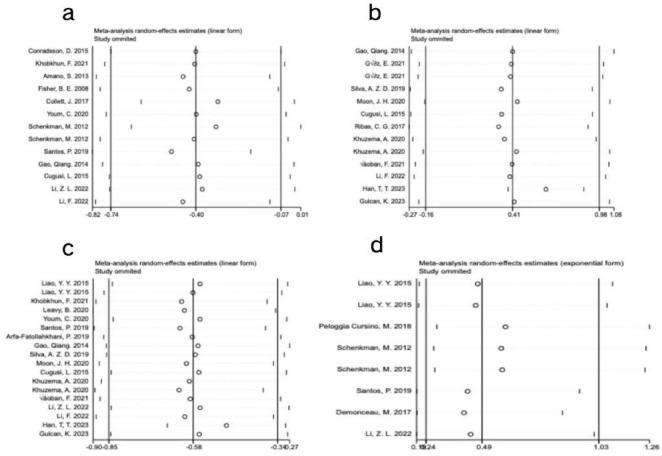
Sensitivity analysis of meta-analysis on the effect of exercise dose on UPDRS-III, BBS, TUG, and PDQ-39 in PD patients. **(a)** UPDRS-III, **(b)** BBS, **(c)** TUG, **(d)** PDQ-39.

Visual inspection of the funnel plot revealed approximate symmetry on both sides, suggesting no apparent publication bias (Figure 4a). Additionally, Begg’s test (*P* = 0.300) and Egger’s test (*P* = 0.062) provided further evidence of the absence of significant publication bias. Sensitivity analysis, conducted by systematically excluding individual studies (Figure 5a), indicated that no single study had a substantial impact on the overall results, confirming the robustness of the findings.

#### 3.4.2 Balance

**FIGURE 6:**
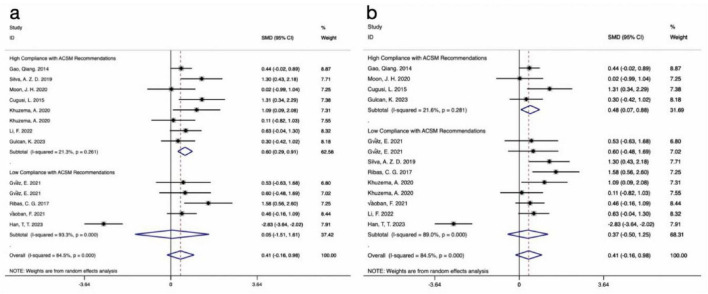
Forest plot of meta-analysis on the effect of exercise dose on BBS in PD patients [ACSM compliance classification criteria: **(a)**, 4/6; **(b)**, 5/6].

The results of 13 studies, including data on the BBS as an outcome measure, were analyzed. A comprehensive assessment of the clinical and methodological characteristics of the included studies was conducted, and a random effects model was used for analysis. The meta-analysis revealed that aerobic exercise intervention could improve the BBS scores in PD patients compared to the control group, but the effect was not statistically significant [SMD, 0.41, (95% CI, −0.16 to 0.98), I^2^ = 84.5%]. A subgroup analysis based on the 4/6 ACSM compliance criterion revealed that, compared to low ACSM compliance, high ACSM compliance in aerobic exercise dose showed a greater improvement in the BBS score for PD patients [High Compliance with ACSM Recommendations: SMD, 0.6, (95% CI, 0.29, 0.91), I^2^ = 21.3%; Low Compliance with ACSM Recommendations: SMD, 0.05, (95% CI, −1.51, 1.61), I^2^ = 93.3%] (Figure 6a). A subgroup analysis based on the 5/6 ACSM compliance criterion also showed that high ACSM compliance in aerobic exercise dose resulted in greater improvement in the BBS score for PD patients [High Compliance with ACSM Recommendations: SMD, 0.48, (95% CI, 0.07, 0.88), I^2^ = 21.6%; Low Compliance with ACSM Recommendations: SMD, 0.37, (95% CI, −0.50, 1.25), I^2^ = 89%] (Figure 6b).

Visual inspection of the funnel plot indicated approximate symmetry on both sides, suggesting no apparent publication bias (Figure 4b). Furthermore, Begg’s test (*P* = 0.855) and Egger’s test (*P* = 0.783) provided additional evidence of the absence of significant publication bias. Sensitivity analysis, conducted by systematically excluding individual studies (Figure 5b), indicated that no single study substantially influenced the overall results, confirming the robustness of the findings.

#### 3.4.3 Mobility

**FIGURE 7:**
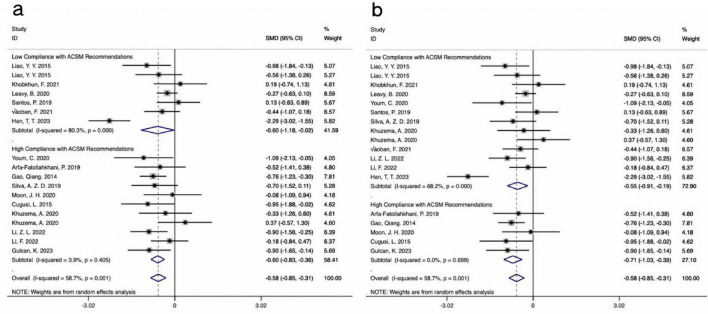
Forest plot of meta-analysis on the effect of exercise dose on TUG in PD patients [ACSM compliance classification criteria: **(a)**, 4/6; **(b)**, 5/6].

The results from 18 studies, incorporating data on the TUG test as an outcome measure, were analyzed. A comprehensive assessment of the clinical and methodological characteristics of the included studies was conducted, and a random effects model was used for analysis. The meta-analysis demonstrated that aerobic exercise intervention could more effectively improve the TUG scores in PD patients compared to the control group [SMD, −0.58, (95% CI, −0.85 to −0.31), I^2^ = 58.7%]. A subgroup analysis based on the 4/6 ACSM compliance criterion revealed that the SMD values for high ACSM compliance and low ACSM compliance were equal [High Compliance with ACSM Recommendations: SMD, −0.60, (95% CI, −0.83, −0.36), I^2^ = 3.9%; Low Compliance with ACSM Recommendations: SMD, −0.60, (95% CI, −1.18, −0.02), I^2^ = 80.3%] (Figure 7a). However, a subgroup analysis based on the 5/6 ACSM compliance criterion showed that, compared to low ACSM compliance, high ACSM compliance in aerobic exercise dose resulted in greater improvement in the TUG score for PD patients [High Compliance with ACSM Recommendations: SMD, −0.71, (95% CI, −1.03, −0.39), I^2^ = 0.00%; Low Compliance with ACSM Recommendations: SMD, −0.55, (95% CI, −0.91, −0.19), I^2^ = 68.2%] (Figure 7b).

Visual inspection of the funnel plot indicated approximate symmetry on both sides, suggesting no apparent publication bias (Figure 4c). Furthermore, Begg’s test (*P* = 0.449) and Egger’s test (*P* = 0.754) provided additional evidence of the absence of significant publication bias. Sensitivity analysis, conducted by systematically excluding individual studies (Figure 5C), indicated that no single study substantially influenced the overall results, confirming the robustness of the findings.

#### 3.4.4 Quality of life

**FIGURE 8:**
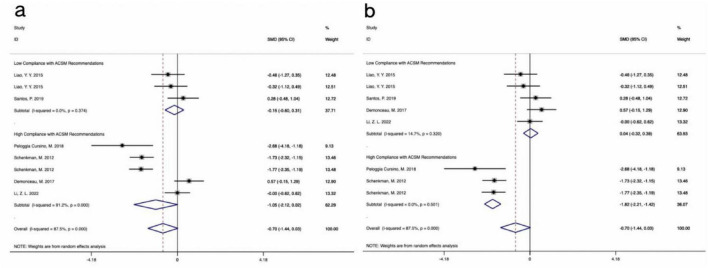
Forest plot of meta-analysis on the effect of exercise dose on PDQ-39 in PD patients [ACSM compliance classification criteria: **(a)**, 4/6; **(b)**, 5/6].

The analysis incorporated data from 8 studies that reported QOL outcomes. A comprehensive assessment of the clinical and methodological characteristics of the included studies was conducted, and a random effects model was used for analysis. The meta-analysis revealed that aerobic exercise intervention could improve the QOL scores in PD patients compared to the control group, although the effect was not statistically significant [SMD, −0.70, (95% CI, −1.44 to 0.03), I^2^ = 87.5%]. When the classification criterion was 4/6 ACSM compliance, the aerobic exercise dose with low ACSM compliance showed little to no improvement in the QOL of PD patients, whereas the aerobic exercise dose with high ACSM compliance improved the QOL of PD patients [High Compliance with ACSM Recommendations: SMD, −1.05, (95% CI, −2.12, 0.02), I^2^ = 91.2%, Low Compliance with ACSM Recommendations: SMD, −0.15, (95% CI, −0.60, 0.31), I^2^ = 0.0%] (Figure 8a). When the classification criterion was 5/6 ACSM compliance, a subgroup analysis revealed that, compared to low ACSM compliance, high ACSM compliance in aerobic exercise dose resulted in greater improvement in the QOL of PD patients [High Compliance with ACSM Recommendations: SMD, −1.82, (95% CI, −2.21, −1.42), I^2^ = 0.00%; Low Compliance with ACSM Recommendations: SMD, 0.04, (95% CI, −0.32, 0.39), I^2^ = 14.7%] (Figure 8b). Moreover, the heterogeneity was significantly reduced after grouping, indicating that ACSM compliance is the main source of heterogeneity.

In assessing publication bias, the funnel plot results were less stable due to the inclusion of fewer than 10 studies. Additionally, heterogeneity in interventions, sample characteristics, and study designs may have influenced the distribution of effect sizes, causing some studies to deviate from expected thresholds (Figure 4d). Therefore, to avoid drawing misleading conclusions, we used Egger’s regression asymmetry test and Begg’s rank correlation test to evaluate publication bias. The results showed no significant publication bias, with Egger’s test (*P* = 0.910) and Begg’s test (*P* = 0.902) both indicating non-significance. Sensitivity analysis using one-by-one exclusion (Figure 5d) revealed no single study had a significant impact on the overall results, demonstrating the robustness of the findings. However, given the small number of included studies and the limited statistical power, publication bias cannot be entirely ruled out, so the bias assessment results should be interpreted with caution.

## 4 Discussion

This systematic review and meta-analysis investigated the impact of aerobic exercise dose on motor function, balance, mobility, and QOL in patients with PD. The study included a comprehensive assessment of exercise modes from previous relevant research and incorporated an analysis of exercise intensity, frequency, and duration in the included studies. The research conducted efficacy analysis of aerobic exercise dose based on compliance with the ACSM recommendations. This study not only deepens the discussion of previous research findings (Cui et al., 2023) but also further validates the efficacy of aerobic exercise for patients with PD (Zhen et al., 2022). Additionally, it provides valuable insights for determining the optimal aerobic exercise dose in the treatment of PD patients.

Firstly, this study delves deeper into previous research. Prior studies have discussed the impact of exercise dose on patients with PD, where compliance with the exercise dose recommended by the ACSM was assessed. Subsequently, a comparison was made between the effects of high compliance and low compliance with ACSM exercise dose on PD patients. However, further refinement is needed in the assessment of ACSM exercise dose (Cui et al., 2023). ACSM-recommended exercise interventions include aerobic exercise, resistance exercise, and flexibility exercise, each with detailed descriptions of recommended exercise dose (Garber et al., 2011). However, in this study, descriptions of exercise dose for PD patients in randomized controlled trials were either incomplete or could only be attributed to one type of exercise intervention. This deficiency in the assessment of exercise dose compliance with ACSM recommendations highlights the need for a more comprehensive evaluation. Therefore, in this study, we specifically assess compliance with ACSM recommendations for aerobic exercise dose and explore the optimal dose and frequency of aerobic exercise to provide a more comprehensive and effective treatment approach for PD patients.

Secondly, regarding aerobic exercise intervention strategies, previous systematic reviews and meta-analyses have discussed the effects of various forms of aerobic exercise on PD patients. Studies by Lai et al. (2022), Suárez-Iglesias et al. (2022), and others suggest that Baduanjin and yoga are effective methods for improving motor symptoms in PD patients. Research by Wu et al. (2022), and Li et al. (2021) found a correlation between exercise intensity and symptom improvement in PD patients, and they suggested that future research should further standardize exercise programs. Additionally, studies focusing on interventions such as dance (Carapellotti et al., 2020), treadmill exercise (Luna et al., 2020), and home-based exercise (Flynn et al., 2019) have shown positive effects. Several network meta-analyses have compared the efficacy of different exercise modalities s (Lorenzo-García et al., 2023; Álvarez-Bueno et al., 2023; Zhang et al., 2023). Based on previous research, it is clear that aerobic exercise can improve symptoms in PD patients, but there is a lack of clear exercise dosage guidelines. Therefore, we focused our research on evaluating aerobic exercise dosage, using motor function, balance, mobility, and QOL as outcome measures to ensure the objectivity and practicality of the results, with the aim of identifying the optimal aerobic exercise dosage for PD patients.

Through this study, we found that compared to the control group or conventional treatment, aerobic exercise has a beneficial effect on Motor function, Mobility, Balance, and QOL in patients with PD. These findings align with the results of Jankovic, (Zhen et al., 2022), and others in “A systematic review and meta-analysis on effects of aerobic exercise in people with PD,” as well as supporting numerous previous experimental studies (Passos-Monteiro et al., 2020; Mak and Wong-Yu, 2021; Soke et al., 2021), affirming the effectiveness of aerobic exercise in improving PD symptoms. In our study, although all four indicators showed improvement, the enhancements in Balance and QOL were not statistically significant when compared to Motor function and Mobility. This is consistent with the findings of de Oliveira et al. (2021), where aerobic exercise did not significantly improve the QOL of PD patients. However, other studies by Franzoni et al. (2018), Soke et al. (2021), Bang and Shin (2017), involving interventions such as treadmill training and Nordic walking, demonstrated that aerobic exercise can positively impact Balance, QOL, and other indicators in PD patients. Therefore, further experiments are still needed to validate these results.

We analyzed the outcome effects of the intervention measures in each study. The analysis revealed that one study, which used Tai Chi as an intervention, did not result in a significant improvement in UPDRS-III scores. This study’s intervention was rated as having low ACSM compliance (Amano et al., 2013). In contrast, in other studies, after a period of intervention, the outcome measures showed positive improvements. Based on this analysis, we find it difficult to determine whether ACSM compliance affects the symptoms of PD patients. Therefore, we compared the differences between high ACSM compliance exercise interventions and those with low or uncertain ACSM compliance.

In the subgroup analysis, when using 4/6 ACSM compliance as the classification criterion, exercise interventions with high ACSM compliance showed greater improvement in PD patients for UPDRS-III (−0.79 vs −0.18), BBS (0.60 vs 0.05), and QOL (−1.05 vs −0.15), but had no effect on TUG (−0.60 vs −0.60). This suggests that under appropriate exercise dose, interventions with higher ACSM compliance may produce more significant effects. However, this finding is partially inconsistent with our previous study, where the improvement in BBS with high ACSM compliance in the comprehensive assessment of exercise types was not significant (Cui et al., 2023). Considering this and the earlier observation that the improvement in Balance and QOL indicators with aerobic exercise was not significant, it appears that aerobic exercise with high ACSM compliance is more advantageous. Comparing the differences in effect size (SMD), the most prominent improvement was observed in QOL (0.9) with high ACSM compliance, followed by UPDRS-III (0.61), and finally BBS (0.55). This suggests that, from the perspective of improving QOL in PD patients, utilizing aerobic exercise with high ACSM compliance is more beneficial.

To ensure the reliability of the results, we conducted a re-evaluation analysis using the 5/6 ACSM compliance criterion. Similar to the results using the 4/6 ACSM compliance classification, high ACSM compliance in exercise interventions showed greater improvement in PD patients for UPDRS-III (−0.95 vs −0.38), BBS (0.48 vs 0.37), and QOL (−0.7 vs 0.04) compared to low ACSM compliance in aerobic exercise dose. Additionally, for TUG (−0.71 vs −0.55), there was a difference compared to the 4/6 ACSM compliance classification, with high ACSM compliance showing better improvement in TUG than low ACSM compliance.

ACSM recommendations encompass aerobic exercise, resistance training, and flexibility exercises. This study primarily analyzed aerobic exercise types because, among the various exercise therapies for PD patients, aerobic exercise has been the most extensively researched and is considered the optimal choice for improving overall health over a lifetime (Voss et al., 2011; Zhen et al., 2022). In the assessment mechanism for aerobic exercise dose, providing a detailed description of the exercise prescription is crucial for precisely determining the reasonable range of the dose. ACSM’s recommended aerobic exercise dose includes descriptions of exercise frequency, intensity, and duration. Although the included randomized controlled trials reported the weekly frequency and duration of aerobic exercise interventions, the descriptions of exercise intensity were not consistently comprehensive. The lack of clear descriptions of exercise intensity in 15 studies led to their classification as NR or Ind. tail. Despite our efforts to ensure objectivity in the assessment, this limitation introduces some degree of error in the evaluation of ACSM compliance. Due to certain limitations in the evaluation of exercise dosage in this study, there may be a bias in the results. In future practical applications, individualized treatment should be provided for patients, with adjustments made within the range of reasonable exercise prescription recommendations.

Furthermore, there is unavoidable heterogeneity and potential publication bias among the included studies. Firstly, the heterogeneity of intervention protocols: Although all 29 studies evaluated aerobic exercise, they included various exercise forms such as balance training, exercise games, Tai Chi, dance, treadmill exercise, and home exercise. This might result in relatively high heterogeneity among studies, and the same issue may be present within subgroups. Differences also exist in specific aerobic exercise protocols, such as intensity, duration, frequency, and type of exercise. This variability introduces heterogeneity, making direct comparison of the study results challenging. Secondly, participant characteristic differences: The characteristics of study participants, such as age, disease severity, Parkinson’s disease duration, and baseline fitness levels, also contribute to heterogeneity. These factors might affect individuals’ responses to aerobic exercise, leading to variations in outcomes. There are also differences in outcome measurement tools: The included studies may have used different tools or scales to measure motor function, balance ability, mobility, and quality of life. This lack of standardization complicates the comparison and synthesis of results.

In each study, there is a potential risk of bias, and factors identified as having unclear or high bias may impact the final estimation of intervention effects. Firstly, there is a positive results bias: Studies with positive or significant results are more likely to be published, while studies with negative or non-significant results may be underestimated or unpublished. This could lead to an overestimation of the true effect of aerobic exercise on PD outcomes. Secondly, selective reporting: Some studies may selectively report results showing significant improvement while ignoring or underreporting non-significant changes. Selective reporting can distort the overall findings of the systematic review. Lastly, in this review, the overall bias results are more likely to be influenced by the blinding of participants and personnel, followed by the allocation and outcome blinding. During the data extraction process for chart results, although we strived to minimize errors, complete avoidance was challenging.

## 5 Conclusion

Through a systematic review and meta-analysis of studies on aerobic exercise interventions, we found that aerobic exercise may have a positive effect on motor function and mobility in patients with PD. Therefore, aerobic exercise holds potential value as an intervention for improving the clinical symptoms of PD patients. However, the impact on balance and QOL remains uncertain and requires further research for validation. In the subgroup analysis, when classified by compliance to ACSM recommendations (4/6 and 5/6 compliance), we found that higher compliance to aerobic exercise interventions may be more advantageous in improving motor function, balance, mobility, and quality of life in PD patients compared to lower compliance. However, it is important to note that the reliability of this finding remains uncertain due to the high level of heterogeneity between studies. In conclusion, given the variations in aerobic exercise dosage, duration, and intervention methods across the studies, as well as the unexplored potential sources of heterogeneity, the results of this study should be interpreted with caution. Future research is needed to further verify these conclusions through meta-regression analyses, focusing on more precise dose-response relationships and other possible influencing factors, such as the duration of interventions.

## Data Availability

The original contributions presented in the study are included in the article/Supplementary material, further inquiries can be directed to the corresponding author.
