# Supplementary material for: The impact of aerobic exercise dose based on ACSM recommendations on patients with Parkinson’s disease: a systematic review and meta-analysis of randomized controlled trials
Source: Front Aging Neurosci. 2024 Oct 3;16:1419643. doi: 10.3389/fnagi.2024.1419643 (PMC11487601; doi:10.3389/fnagi.2024.1419643)
Supplement: Supplementary file 1 [file Table_1.docx]

Supplementary Material

# Supplementary Figures and Tables

**Appendix 1** Search Strategy

| Pubmed | | |
| --- | --- | --- |
| #1 | Search: "Parkinson Disease"[Mesh] Sort by: Most Recent | 80374 |
| #2 | Search: ((((((((((Idiopathic Parkinson's Disease[Title/Abstract]) OR (Lewy Body Parkinson's Disease[Title/Abstract])) OR (Parkinson's Disease, Idiopathic[Title/Abstract])) OR (Parkinson's Disease, Lewy Body[Title/Abstract])) OR (Parkinson Disease, Idiopathic[Title/Abstract])) OR (Parkinson's Disease[Title/Abstract])) OR (Idiopathic Parkinson Disease[Title/Abstract])) OR (Lewy Body Parkinson Disease[Title/Abstract])) OR (Primary Parkinsonism[Title/Abstract])) OR (Parkinsonism, Primary[Title/Abstract])) OR (Paralysis Agitans[Title/Abstract]) | 105297 |
| #3 | #1 OR #2 | 124713 |
| #4 | Search: "Exercise"[Mesh] Sort by: Most Recent | 242211 |
| #5 | Search: ((((((((((((Exercises[Title/Abstract]) OR (Sports[Title/Abstract])) OR (Physical Activity[Title/Abstract])) OR (Motor Activity[Title/Abstract])) OR (Training[Title/Abstract])) OR (endurance training[Title/Abstract])) OR (Tai Chi[Title/Abstract])) OR (yoga[Title/Abstract])) OR (Balance[Title/Abstract])) OR (Resistance[Title/Abstract])) OR (Flexibility[Title/Abstract])) OR (Cardiovascular[Title/Abstract])) OR (Aerobic[Title/Abstract]) | 2504823 |
| #6 | #4 OR #5 | 2613131 |
| #7 | Search: ((((Randomized controlled trial[Publication Type]) OR (controlled clinical trial[Publication Type])) OR (randomized[Title/Abstract])) OR (placebo[Title/Abstract])) OR (randomly[Title/Abstract]) | 1309531 |
| #8 | #3 AND #6 AND #7 | 1439 |
| embase | | |
| #1 | 'parkinson disease'/exp OR 'parkinson disease' OR (parkinson AND ('disease'/exp OR disease)) OR 'idiopathic parkinsons disease':ab,ti OR 'lewy body parkinsons disease':ab,ti OR 'parkinsons disease, idiopathic':ab,ti OR 'parkinsons disease, lewy body':ab,ti OR 'parkinson disease, idiopathic':ab,ti OR 'parkinsons disease':ab,ti OR 'idiopathic parkinson disease':ab,ti OR 'lewy body parkinson disease':ab,ti OR 'primary parkinsonism':ab,ti OR 'parkinsonism, primary':ab,ti OR 'paralysis agitans':ab,ti | 245031 |
| #2 | ('exercise'/exp OR exercise OR exercises:ab,ti OR sports:ab,ti OR 'physical activity':ab,ti OR 'motor activity':ab,ti OR training:ab,ti OR 'endurance training':ab,ti OR 'tai chi':ab,ti OR yoga:ab,ti OR balance:ab,ti OR resistance:ab,ti OR flexibility:ab,ti OR cardiovascular:ab,ti OR aerobic:ab,ti) AND ([controlled clinical trial]/lim OR [randomized controlled trial]/lim) | 195609 |
| #3 | #1 AND #2 | 1897 |
| web of science | | |
| #1 | (((((((((((TS=(Parkinson Disease)) OR TS=(Idiopathic Parkinson's Disease)) OR TS=(Lewy Body Parkinson's Disease)) OR TS=(Parkinson's Disease, Idiopathic)) OR TS=(Parkinson's Disease, Lewy Body)) OR TS=(Parkinson Disease, Idiopathic)) OR TS=(Parkinson's Disease)) OR TS=(Idiopathic Parkinson Disease)) OR TS=(Lewy Body Parkinson Disease)) OR TS=(Primary Parkinsonism)) OR TS=(Parkinsonism, Primary)) OR TS=(Paralysis Agitans) | 147845 |
| #2 | (((((((((((((TS=(Exercise)) OR TS=(Exercises )) OR TS=(Sports)) OR TS=( Physical Activity )) OR TS=(Motor Activity)) OR TS=( Training)) OR TS=(endurance training)) OR TS=( Tai Chi )) OR TS=(yoga )) OR TS=( Balance )) OR TS=(Resistance)) OR TS=(Flexibility )) OR TS=(Cardiovascular )) OR TS=(Aerobic) | 5818661 |
| #3 | ((((TS=(Randomized controlled trial )) OR TS=(controlled clinical trial )) OR TS=( randomized )) OR TS=(placebo )) OR TS=( randomly) | 1603217 |
| #4 | #1 AND #2 AND #3 | 2703 |
| Cochrane | | |
| #1 | (Parkinson Disease):ti,ab,kw OR (Idiopathic Parkinson's Disease):ti,ab,kw OR (Lewy Body Parkinson's Disease):ti,ab,kw OR (Parkinson's Disease, Idiopathic):ti,ab,kw OR (Parkinson's Disease, Lewy Body):ti,ab,kw | 12188 |
| #2 | (Parkinson Disease, Idiopathic):ti,ab,kw OR (Parkinson's Disease):ti,ab,kw OR (Idiopathic Parkinson Disease):ti,ab,kw OR (Lewy Body Parkinson Disease):ti,ab,kw OR (Primary Parkinsonism):ti,ab,kw | 12319 |
| #3 | (Parkinsonism, Primary):ti,ab,kw OR (Paralysis Agitans):ti,ab,kw | 4044 |
| #4 | #1 OR #2 OR #3 | 12320 |
| #5 | (Exercise):ti,ab,kw OR (Exercises):ti,ab,kw OR (Sports):ti,ab,kw OR (Physical Activity):ti,ab,kw OR (Motor Activity):ti,ab,kw | 182622 |
| #6 | (Training):ti,ab,kw OR (endurance training):ti,ab,kw OR (Tai Chi):ti,ab,kw OR (yoga):ti,ab,kw OR (Balance):ti,ab,kw | 177633 |
| #7 | (Resistance):ti,ab,kw OR (Flexibility):ti,ab,kw OR (Cardiovascular):ti,ab,kw OR (Aerobic):ti,ab,kw | 192182 |
| #8 | #5 OR #6 OR #7 | 429673 |
| #9 | (Randomized controlled trial):pt OR (controlled clinical trial):pt OR (randomized):ti,ab,kw OR (placebo):ti,ab,kw OR (randomly):ti,ab,kw | 1313648 |
| #10 | #4 AND #8 AND #9 | 4267 |
